# Evaluation of the Effectiveness of an Interdisciplinary Preventive Oral Hygiene Program for Children with Congenital Heart Disease

**DOI:** 10.3390/ijerph18073497

**Published:** 2021-03-28

**Authors:** Nelly Schulz-Weidner, Thushiha Logeswaran, Christian Jux, Maximiliane Amelie Schlenz, Norbert Krämer, Julia Camilla Bulski

**Affiliations:** 1 Dental Clinic—Department of Pediatric Dentistry, Justus Liebig University, Schlangenzahl 14, 35392 Giessen, Germany; norbert.kraemer@dentist.med.uni-giessen.de (N.K.); julia.c.bulski@dentist.med.uni-giessen.de (J.C.B.); 2 Department of Pediatric Cardiology and Congenital Heart Disease, Pediatric Heart Centre, Justus Liebig University, Giessen Feulgenstrasse 12, 35394 Giessen, Germany; thushiha.logeswaran@paediat.med.uni-giessen.de (T.L.); christian.jux@paediat.med.uni-giessen.de (C.J.); 3 Dental Clinic—Department of Prosthodontics, Justus Liebig University, Schlangenzahl 14, 35392 Giessen, Germany; maximiliane.a.schlenz@dentist.med.uni-giessen.de

**Keywords:** oral hygiene program, congenital heart disease, oral health status, pediatric dentistry, interdisciplinary study

## Abstract

It is recognized that children with congenital heart disease (CHD) are predisposed to having poorer oral health. Therefore, the purpose of this study was to evaluate the effectiveness of an interdisciplinary preventive oral hygiene program (POHP) for children with CHD. The aim was the reduction of the incidence of dental caries, as well as improvement of oral hygiene. The total number of participants in this study was 107 children with CHD aged between two to six years. At baseline, these children were compared to a healthy control group (HCG) of 101 children of similar age from five preschools in Giessen, Germany. All examinations were carried out before the introduction of a standardized POHP. The *Quigley/Hein* Plaque- (QHI), *Silness/Loe* Gingival- (GI) and Gingival Hyperplasia Index (GHI) were determined. Starting with baseline, the described procedures were repeated in the CHD group during two follow-ups after three and six months. In the first examination, compared to controls, CHD children showed a significantly (*p* < 0.05) poorer oral hygiene (QHI: 2.6; GI: 0.3; GHI: 0.2). All oral hygiene parameters (QHI, GI, GHI) of the CHD group improved significantly over the whole period of the preventive program (*p* < 0.05). These results demonstrated an improvement in CHD children involved in a standardized POHP. The data with regard to the general health of these risk patients, including prevention of endocarditis, demonstrate the necessity of an interdisciplinary approach between pediatric cardiologists, pediatricians and dentists.

## 1. Introduction

Around 6500 to 8000 children per year in Germany are born with a congenital heart disease (CHD) [1,2]. These children are predisposed to develop oral diseases such as gingivitis due to poor oral hygiene and concomitant caries [3,4,5]. In addition, the prevalence of tooth decay is significantly higher than in healthy children [3,4,5,6,7,8,9]. Studies demonstrated that this impairment of oral health has dangerous systemic effects, especially episodes of increased bacteremia in one third of these children [7,10]. Regarding the risk of endocarditis and even dental sepsis, also untreated carious teeth, there seems to be a significant problem for children with congenital heart disease.

Therefore, early dental intervention is a suggested tool to optimize oral health, including the prevention of infective endocarditis [11,12]. In the group of children with cardiac disease, preventive measures are recommended as early as possible [13,14]. A study in Norway described the first published prophylaxis program for children with heart disease and indicated better oral hygiene with reduced gingival bleeding and a lower number of untreated dentine lesions in these children compared after the second follow-up [10]. Standardized oral health programs have been described in the literature as very effective for patients with CHD, but mostly investigate the oral health status at one point only and not over a longer period [4,6,14,15,16,17,18]. Besides a lack of awareness of the importance of oral health among parents of CHD children, other aspects seem to have an influence on the possible prevention of caries and gingival diseases. For example, the time required for frequent appointments with the cardiologist seems to favor the neglect of dental visits and regular dental check-ups [19]. This indicates that for an optimized oral hygiene an interdisciplinary cooperation between pediatric cardiologists, pediatricians and dentists is necessary in order to improve oral and dental health for children with CHD [13,18,19,20,21].

Therefore, the aim of this study was to evaluate the effectiveness of an interdisciplinary preventive oral hygiene program (POHP) in children with CHD over a period of six to twelve months. Firstly, it was determined whether these children show a difference in dental and oral health compared to a control group of healthy children (HCG) and the need for treatment measurements was analyzed. Secondly, an already established and standardized oral health prevention concept of the Association for Youth Dental Care in Hessen, Germany (LAGH) [22,23,24,25,26], which is regularly performed in institutions among healthy preschool children, was conducted in the CHD group in the context of their cardiologic control appointments.

## 2. Materials and Methods

From February 2018 to August 2019, clinical investigations took place at the Center for Pediatrics (Department of Pediatric Cardiology, Justus Liebig University, Giessen, Germany), as well as in five preschools in and nearby Giessen.

### 2.1. Subjects and Setting

In the study, 107 (45 girls, 62 boys) children with congenital heart defects (CHD) and 101 (45 girls, 46 boys) healthy children (HCG) aged between two and six years participated. Only children with CHD, with at least one surgical heart intervention were included. The healthy control group (HCG) consisted of children with a healthy general condition or without a significant handicap (maximum ASA class I).

For the admission of a child, an informed consent presented and signed by the parents or legal guardians was necessary. The study was realized in accordance with the guidelines of the Declaration of Helsinki and approved by the local ethics committee of the Department of Medicine, Justus Liebig University Giessen (Ref. no. 186/17).

For calibration of the main examiner (J.C.B.), Cohen’s kappa (κ) was used for the measurement of inter- and intra-examiner reliability. The intensity of correspondence of the examiner was almost perfect (κ = 0.83, weighted κ = 0.89).

### 2.2. Dental and Gingival Examination

All examinations were conducted with a plane mouth mirror. The dental status was recorded by trained, calibrated clinicians following the WHO standard criteria [27]. Caries status was assessed by the decayed, missing and filled teeth (dmf-t index) [28]. In addition, initial carious teeth (i-t) were observed. For simplification of the survey differentiation between enamel and dentine caries was applied. No radiographs were taken. After the baseline examination, the gingival alterations were determined using the *Quigley/Hein* Plaque Index (QHI), the *Silness/Loe* Gingival Index (GI) and the Gingival Hyperplasia Index (GHI) in two follow-ups (FU1 and FU2). Regarding the QHI, the coronal facial surfaces were stained with a plaque elevator (Miradent, Hager and Werken GmbH and Co KG, Duisburg, Germany). After the patient had been rinsed thoroughly, the remaining stained areas were identified by areas classified on the six modified Ramfjord teeth (55 instead of 16, 61 instead of 21, 64 instead of 21, 75 instead of 36, 81 instead of 41 and 84 instead of 44) [29,30,31,32].

### 2.3. Preventive Oral Hygiene Program (POHP)

A preventive oral hygiene program (POHP) with a prophylaxis schedule, already established in preschools throughout Germany, was used according to the concept of Association for Youth Dental Care in Hessen, Germany (LAGH) [22,23,24,25,26]. The participating preschools were already experienced in this well-known concept, which is regularly supported by a dentist. This support is usually divided into three parts. The first part includes oral hygiene demonstrations and motivation. In the second part, oral hygiene measures regarding tooth brushing and healthy nutrition are checked and trained, including motivation. In this context, given the age of the participating children, besides the parental awareness and attention to oral health, an important aspect of parental work is the recommendation of the LAGH due to insufficient motor maturity of the child, to brush the children’s teeth with a manual toothbrush until the child can write fluently. The third part is a check-up including a visit to the dentist. In this process the preschool employees are routinely integrated (training courses for the educators) [22,23,24,25,26].

Based on this concept, the children with CHD were instructed in the same standardized oral hygiene program. After the initial examination, two follow-up appointments were made at intervals of three to six months, at which the children were reexamined and additionally remotivated in their tooth brushing behavior. With the initial examination, the intensive care program of the children with cardiac disease was started by the attending dentist. The examination of the CHD group was carried out as part of the regular cardiological recall appointment. According to their age, the children and their parents were instructed in the KAI^plus^ tooth brushing technique (K: Kaufläche/chewing surface; A: Außenfläche/external surface; I: Innenfläche/inner surface) [25] by the dentist calibrated for this purpose. Tooth brushing was performed twice daily (after breakfast and after the last meal in the evening) with a fluoride-containing toothpaste (500 ppm). Dental flossing was not used because children between the ages of two and six years do not yet have the necessary coordinative and motor skills for correct application. Tap water fluoridation was not taken into account, as it is not considered in caries prevention due to its negligible amount [33]. In addition, an almost “sugar-free day” was proposed, which started after brushing the teeth in the morning [22]. During this period, chewable, natural foods could be offered as a snack and beverages without sugar (e.g., mineral water, unsweetened teas). A minimum 16-h regeneration period, consisting of 12 h after dinner and night’s rest and the four hours of the sugar-free morning, was provided for the enamel by the saliva.

Depending on the interval of the recall examination, the first follow-up examination (FU1) took place in the Department of Pediatric Cardiology after three to six months. Again, after an interval of three to six months, the final examination (FU2) followed. During the POHP, the mentioned parameters (QHI, GI, GHI) were reevaluated in the first and second follow-up. Furthermore, every single examination included special oral hygiene training with instructions for each child and their parents, as well as practicing tooth brushing together, including remotivating for the procedure.

The oral hygiene status of the HCG was taken up uniquely in a baseline investigation, since the children of Hessian preschools are already integrated into this standardized prophylaxis program of LAGH. The dental parameters were collected according to the baseline examination of the CHD group. Again, the KAI^plus^ system was verified in small groups during tooth brushing. An age-appropriate children’s toothbrush and a fluoride-containing children’s toothpaste (500 ppm) were also used, which were already available in the preschools. The remotivation in the sense of a follow-up was carried out by the parents and group supervisors after half a year in a written form using information material (parental work), which was made available by the LAGH.

### 2.4. Statistical Analysis

The data analysis was exploratory in nature. The data acquisition and the construction of graphics was accomplished with the DOS-based program dBASE IV (Borland, Austin, TX, USA) and Microsoft^®^ Excel (Office Version 2011, Microsoft Cooperation, Redmond, WA, USA). The statistical analysis of data was performed using the software package SPSS^®^ for Windows (version 25.0, IBM Corporation, Armonk, New York, NY, USA). For categorical characteristics, the absolute and relative frequencies were calculated, and the quantitative (ordinal scaled) data were described by the median, mean value, minimum and maximum. The differences between the two groups (CHD and HCG) concerning their mean age and mean plaque values were evaluated using the *t*-test for independent samples (confidence interval 95%). Using the Chi-squared test (χ^2^ test), the gender distribution could be calculated. In addition, for comparison of the mean GI and GHI of group CHD and HCG at baseline the Mann-Whitney-U test was applied. The development of the parameters from baseline to the second follow-up was compared with the *t*-test and Wilcoxon test. The level of significance was set at *p* < 0.05.

## 3. Results

At baseline, 107 children with congenital heart disease (CHD) and 101 healthy children (HCG) participated in the study. The mean age for all CHD children was 4.63 ± 1.46 years compared to the HCG group (4.4 ± 1.21), without a statistical difference between both groups (*t*-test, *p* > 0.05). Among the CHD group, various cardiac defects of all severities (light, moderate, severe [34]) were included as described before. Sixty-five percent of children were affected by severe CHD. Furthermore, 11 children with rare syndromes were also included in our study, e.g., trisomy 21, 22q11, Williams-Beuren-, Barth-, Turner- or Charge syndrome.

### 3.1. Caries Experience

At the start of the study, 71% of CHDs had no caries experience (dmf-t = 0). These children did not have decayed, filled or extracted first teeth due to caries before. The prevalence of no caries history in the HCG with 87.1% was significantly higher compared to the CHD group (Mann-Whitney-U test, *p* < 0.01). After all follow-ups, a significant decrease of 8.9% in caries prevalence in the CHD group could be observed (McNemar test, *p* < 0.05).

**Figure 1:**
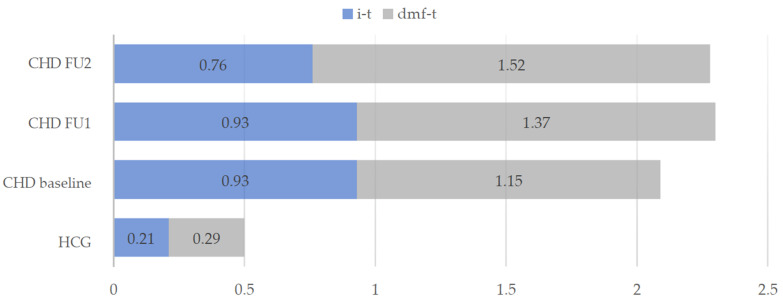
Mean dmf-t values and initial carious teeth (i-t) in the intervention group (CHD) and healthy control group (HCG) at baseline and during the two follow-ups (FU1 and FU2) of the CHD group.

Figure 1 shows the distribution of the mean dmf-t values of HCG and of the CHD group throughout the three appointments. In the group of CHD children an average number of one deciduous tooth (0.93 ± 1.90) was affected by an initial carious lesion (i-t). In the HCG, the mean i-t value (0.21 ± 0.77) was significantly lower (Mann-Whitney-U test, *p* < 0.001).

Children with cardiac disease showed significantly increased treatment needs compared to the healthy children. The proportion of 17.8% untreated carious lesions in the first dentition of CHD children was higher in comparison to the HCG (6.9%). In the course of the prophylaxis program, an increase of the dmf-t value within the CHD group could be observed from the baseline examination to the final results with a significant difference between the two follow-ups (Wilcoxon test, *p* < 0.05), whereby the extraction of carious deciduous teeth (m-t) increased significantly (*p* < 0.05) from the first (FU1) to the second (FU2) follow-up (Table 1).

### 3.2. Oral Hygiene Parameters

The results of our study showed a poorer oral hygiene condition within the children with congenital heart disease (CHD) compared to the healthy control group (HCG) at the time of baseline. In general, after two follow-ups in the CHD group all oral hygiene parameters improved.

**Figure 2:**
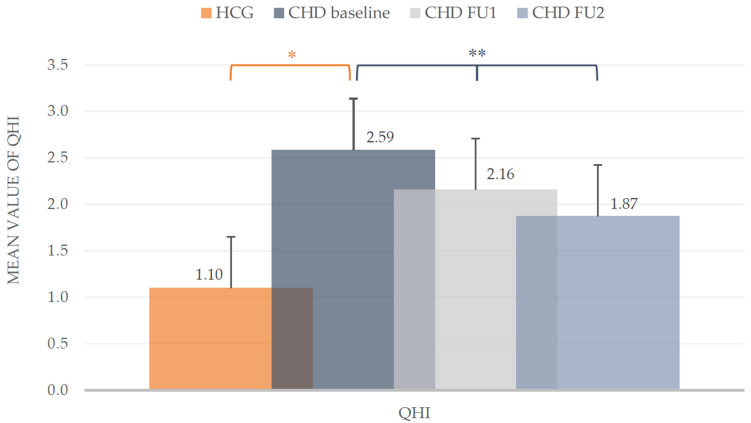
*Quigley/Hein* Plaque Index (QHI). Mean values including standard deviation of the intervention group (CHD) and healthy control group (HCG) at baseline and during the two follow-ups (FU1 and FU2) of the CHD group. * Mann-Whitney-U test (*p* < 0.05), ** *t*-test (*p* < 0.05).

At baseline the mean plaque values (QHI) in the CHD group were much higher compared to the HCG (2.59 ± 0.81 vs. 1.10 ± 0.55). The mean values of both groups differed statistically (*t*-test, *p* < 0.001). During the course of the POHP, a clear reduction of the QHI in CHD children could be achieved. From baseline (2.59 ± 0.81) to the first follow-up (2.16 ± 0.66) the mean value decreased significantly about 16.6% (*t*-test, *p* < 0.001). At the second follow-up the mean QHI dropped to 1.87 ± 0.64 with a significant reduction (*t*-test, *p* < 0.001). Overall, the plaque accumulation in the CHD group (Figure 2) was optimized around one third (27.8%).

**Figure 3:**
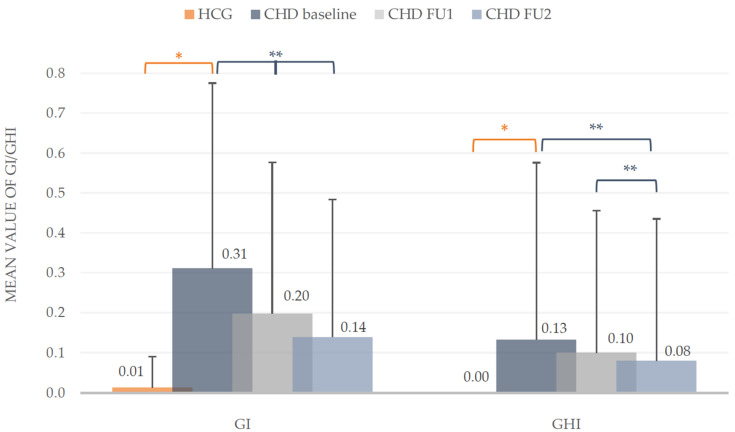
*Silness/Loe* Gingival Index (GI) and Gingival Hyperplasia Index (GHI). Mean values including standard deviation of the intervention group (CHD) and healthy control group (HCG) at baseline and during the two follow-ups (FU1 and FU2) of the CHD group. * Mann-Whitney-U test (*p* < 0.05), ** Wilcoxon test (*p* < 0.05).

At baseline, the examination showed no signs of gingival inflammation and hyperplasia in the HCG (GI: 0.01 ± 0.08; GHI: 0). Compared to the CHD group, these problematic gingival findings differed significantly (Mann-Whitney-U test, *p* < 0.001). During the prophylactic intervention the development of the GI and GHI declined. The GI of the CHD group dropped significantly in comparison to baseline (Wilcoxon test, *p* < 0.001), as well as to the first and second follow-up (Wilcoxon test, *p* < 0.01). At the first follow-up an improvement of the GI about 35.48% and in the end about one half (54.84%) could be observed, as well as in gingival hyperplasia about 38.46%. The findings of the second follow-up for the GHI in the group of children with congenital heart disease (CHD) differed significantly to the mean values of baseline and first follow-up (Wilcoxon test, *p* < 0.01) with a total reduction of 38.46%. The detected reduction of GHI from first to second follow-up was not statistical different (*p* > 0.05, Figure 3).

## 4. Discussion

This study provides evidence that the oral health of children with congenital heart disease (CHD) is significantly poorer compared to healthy controls, which is in accordance with most of the literature [3,5,8,9,11,35]. In our study, we included 11 children with rare syndromes. It can be discussed if these children show a worser dental care ability and may influence the results of the CHD group. However, at this age parents are an important component in oral health optimization, so we decided not to exclude these children from our study.

While in two other studies [17,36] a higher level of caries occurrence in the cardiac disease group was demonstrated, in this study not only the dmf-t increased in CHD children but rather the number of rehabilitated deciduous teeth (f-t) implying that affected children consulted a dentist.

Furthermore, an increased plaque occurrence in the CHD group was determined in good accordance with other studies [3,5,7,18,19,37]. *Sivertsen* et al. found a plaque accumulation in 88.1% on at least one deciduous tooth, frequently affecting the second molars [5]. *Franco* et al. described the same in the primary dentition twice as high as in the permanent dentition in these children [38]. These findings are different to other authors who were not able to confirm these data [4,15,17,38].

Concerning the oral hygiene status, the described different indices (GI, QHI) differed significantly from the healthy controls. The mean values of the Gingival Indices (GI: 0.31 ± 0.46) of the CHD group were significantly higher compared to the controls. These healthy children in the present study showed no signs of gingival inflammation. Moreover, the GHI of 0.13 ± 0.44 in CHD children showed a further oral problem in this vulnerable group of children which is in agreement with other international studies reporting a prevalence of 1.5% [5] and 13.5% [3] for gingival hyperplasia in children with CHD. It is accepted that gingival hyperplasia is mainly caused by dental independent factors (e.g., medication), but could be improved through optimized oral hygiene as demonstrated in a small group of heart transplanted patients [37].

However, the results of our study confirmed that improvement of oral hygiene parameters by a standardized Preventive Oral Hygiene Program (POHP) including continuous dental care with instructions for tooth brushing and remotivation have even a favorable effect on the reduction of inflammation and gingival hyperplasia. We observed an approximately 30% reduction in the average plaque values (QHI) which indicates in our view the better implemented tooth brushing technique. Furthermore, in our group of CHD children the degree of inflammation of the gingiva could be reduced by half. Additionally, in the follow-up a significant minimization of gingival hyperplasia from the initial until the final appointment could be demonstrated. A limitation of our study is the missing follow-up of the control group (HCG). We neglected this aspect because dental care in the preschools is already established in Hessen (Germany) assuming that an improvement of the oral health takes place.

The results of the present study provide important information on the improvement of the oral health status of children with CHD when a standardized POHP has been established. With regard to the improvement of oral health in this high-risk group, regular and ongoing check-ups and motivation of the children in their dental health behavior seem to play a major role. In addition to the necessary treatment of carious lesions, general oral hygiene and the motivation of oral health condition appear to be important. The success of the program in the context of cardiological controls underlines the importance of consultative care in order to identify and treat possible oral health problems at an early stage and, in the best case, to prevent them. Additionally, further prophylactic measurements (fluoridation in children’s toothpaste, varnishes and nutritional guidance) should be considered [39].

This study indicates that the described guarantee of early and continuous dental care is an important aspect for health in these children with congenital heart defects, especially regarding the possible deleterious outcome of an infectious odontogenic endocarditis. However, a clear limitation is that only a single-center-study was conducted. Therefore, it would be desirable if other clinics would implement an interdisciplinary standardized POHP as well. In addition, the study of a longer period would also be interesting in terms of a possible improvement of the general condition due to an improved oral health status.

## 5. Conclusions

Within the limitation of this study, it could be shown that interdisciplinary cooperation between pediatric cardiologists and dentists in a structured preventive oral hygiene program significantly improves the oral health status of children with CHD. Ideally, the dental examination and consultation should be integrated into the pediatric cardiologic diagnostic protocol.

## Figures and Tables

**Table 1:** Dmf-t values of the CHD group: decayed (d), filled (f) and missing (m) deciduous teeth (t). Mean values (M) including standard deviation (SD) at baseline and after the two follow-ups (FU1 and FU2).

CHD	Baseline		FU1		FU2	
	M	SD	M	SD	M	SD
d-t	0.42 ^A^	1.21	0.50 ^A^	1.46	0.42 ^A^	1.23
m-t	0.11 ^A,B^	0.52	0.11 ^A^	0.51	0.20 ^B^	0.68
f-t	0.62 ^A^	1.77	0.75 ^A^	1.92	0.88 ^A^	1.97
dmf-t	1.15 ^A^	2.5	1.37 ^B^	2.65	1.52 ^B^	2.73

^A-A^ No significant difference (Wilcoxon test, *p* > 0.05), e.g., mean value (M) of d-t from FU1 to FU2. ^B-B^ No significant difference (Wilcoxon test, *p* > 0.05), e.g., mean value (M) of dmf-t from FU1 to FU2. ^A-B^ Significant difference (Wilcoxon test, *p* < 0.05), e.g., mean value (M) of m-t from baseline to FU2 and FU1 to FU2.

## Data Availability

The data presented in this study are available on request from the corresponding author.
